# Antimicrobial Activity of Actinobacteria Isolated From Kratom (*Mitragyna speciosa*) Leaves: Secondary Metabolite Profiling and Genome Analysis of *Micromonospora chersina* NRAIS18

**DOI:** 10.1155/ijm/3823241

**Published:** 2026-05-05

**Authors:** Nittaya Pitiwittayakul, Nanthavut Niyomvong, Nisachon Tedsree, Somboon Tanasupawat

**Affiliations:** ^1^ Department of Plant Science, Faculty of Agricultural Innovation and Technology, Rajamangala University of Technology Isan, Nakhon Ratchasima Campus, Nakhon Ratchasima, Thailand, rmuti.ac.th; ^2^ Science Center, Nakhon Sawan Rajabhat University, Nakhon Sawan, Thailand; ^3^ Department of Biology and Biotechnology, Faculty of Science and Technology, Nakhon Sawan Rajabhat University, Nakhon Sawan, Thailand; ^4^ Department of Agricultural Technology, Faculty of Science and Arts, Burapha University, Chanthaburi Campus, Chanthaburi, Thailand, buu.ac.th; ^5^ Department of Biochemistry and Microbiology, Faculty of Pharmaceutical Sciences, Chulalongkorn University, Bangkok, Thailand, chula.ac.th

**Keywords:** actinobacteria, antimicrobial activity, bioactive compound, *Micromonospora*, *Mitragyna speciosa*

## Abstract

Actinobacteria associated with medicinal plants are recognized as prolific sources of novel bioactive compounds. Kratom (*Mitragyna speciosa*) leaves have been reported to contain a diverse array of secondary metabolites with pharmacological activities, including antibacterial, antioxidant, and anti‐inflammatory effects. In this study, 16 actinobacterial isolates were successfully obtained from the leaves of *M. speciosa*. Based on the 16S rRNA gene sequence analysis, the isolates were identified as members of *Streptomyces* and non‐*Streptomyces* genera, including *Micromonospora*, *Pseudonocardia*, *Quadrisphaera*, *Prauserella*, and *Actinomycetospora*. All isolates were screened for antibacterial activity, among which *Micromonospora chersina* NRAIS18 demonstrated notable inhibitory effects against both Gram‐positive and Gram‐negative pathogens. The ethyl acetate crude extract of NRAIS18 exhibited the most potent activity against *Listeria monocytogenes* ATCC 7644, followed by *Pseudomonas aeruginosa* ATCC 27853. Metabolomic profiling using LC‐MS and GC‐MS revealed diverse secondary metabolites in the crude extract, including the isoflavone daidzein, harman, norharman, and soyasaponin Bb; siderophores such as ferrioxamine E and desferrioxamine E; and other volatile bioactive compounds such as fatty acids, isoquinolines, and esters. Several of these metabolites have been reported to possess antibacterial properties. These findings suggest that *M. chersina* NRAIS18 demonstrates significant promise as a source of antibacterial agents for pharmaceutical applications.

## 1. Introduction

Kratom, the common name for *Mitragyna speciosa* (Korth.), is a native plant of Southeast Asia belonging to the *Rubiaceae* family. It grows naturally in several regions, including Thailand [1]. Traditionally, its raw leaves have been consumed for their soothing and analgesic properties. Across Southeast Asia, kratom leaves have also been used to alleviate diarrhea, muscle soreness, and high blood pressure, as well as to enhance stamina [2]. Crude alkaloidal extracts from *M. speciosa* leaves have demonstrated antidiabetic properties by boosting key enzyme activities and enhancing glucose uptake and transport [3]. Juanda et al. [4] reported that the methanolic extracts of kratom leaves exhibited antibacterial activity against *Aeromonas hydrophila* and contained beneficial secondary metabolites, including alkaloids, saponins, tannins, phenolics, steroids, and triterpenoids.

Actinomycetes have been reported as epiphytes inhabiting the phyllosphere or as endophytes residing within the leaf, root, and stem tissues of plants [5, 6]. A wide range of actinobacteria isolated from medicinal plants have shown potential to produce novel compounds with applications in both agriculture and pharmaceuticals [7, 8]. Endophytic actinomycetes associated with medicinal plants such as *Camellia sinensis*, *Mirabilis jalapa*, and *Clerodendrum colebrookianum* have demonstrated broad‐spectrum antimicrobial activity [9, 10]. Epiphyte actinomycetes inhabiting leaf surfaces are considered an important microbial resource because the phyllosphere represents a dynamic habitat where microorganisms can produce metabolites that contribute to plant protection and microbial interactions [11]. Due to its rich phytochemical composition and long history of medicinal use, *M. speciosa* may provide a unique ecological niche that supports diverse microbial communities capable of producing biologically active metabolites [12].

However, studies on microorganisms associated with *M. speciosa* remain limited. Previous research has reported endophytic actinomycetes belonging to the genus *Streptomyces* isolated from the leaves of this plant, including the novel species *Streptomyces mitragynae*, which exhibited antimicrobial potential and diverse secondary metabolites [13]. In addition, endophytic fungi associated with *M. speciosa* have been reported. For example, *Diaporthe* sp. isolated from the twigs of *M. speciosa* produces cytosporone D, an octaketide with antibacterial and antibiofilm activities [14]. These findings highlight *M. speciosa*–associated endophytes as a promising source of biologically active metabolites. Notably, *M. speciosa* itself is well‐known for its rich repertoire of bioactive compounds with potential medicinal value. Therefore, this study is aimed at isolating and identifying actinomycetes from *M. speciosa* leaves, evaluating their antimicrobial activity, and analyzing the secondary bioactive compounds of the most potent strain using gas chromatography–mass spectrometry (GC‐MS) and liquid chromatography–mass spectrometry (LC‐MS) analyses.

## 2. Materials and Methods

### 2.1. Surface Sterilization

Kratom (*M. speciosa* Korth.) leaves were both purchased from local commercial sources and freshly collected from cultivated trees in Nakhon Ratchasima Province, Thailand. The plant materials were transported to the laboratory in sterile containers and processed immediately to ensure freshness. Kratom is no longer classified as a controlled narcotic in Thailand [15], and therefore, no specific collection permit was required for this study. Surface sterilization of *M. speciosa* Korth. leaves was carried out following modified procedures described by Pitiwittayakul et al. [16] and Golinska et al. [8]. The plant tissues were first washed thoroughly under running tap water to remove contaminants and any physical debris. The samples were then cut into small pieces and subjected to a sterilization sequence: immersion in 70% ethanol for 3 min, followed by treatment with 2% fresh sodium hypochlorite solution for 5 min, rinsing with sterile distilled water, a second immersion in 70% ethanol for 30 s, and a final rinse with sterile distilled water three times. To confirm the effectiveness of the sterilization protocol, 100‐*μ*L aliquots of the sterile water from the final rinse were spread on Starch M‐Protein Agar (SMA) (Himedia, India). The plates were incubated at 30°C for 1 week and examined for bacterial growth. For the isolation of epiphytic actinomycetes, surface sterilization was omitted. In this case, *M. speciosa* leaves were only washed with tap water and rinsed with sterile distilled water.

### 2.2. Isolation of Endophytic and Epiphytic Actinomycetes

After surface‐sterilization, the sterilized samples were ground using a sterile mortar and pestle, then serially diluted in 0.85% normal saline solution. Approximately 100 *μ*L of each plant extract dilution (10^−1^, 10^−2^, and undiluted for endophyte isolation; 10^−4^–10^−6^ for epiphyte isolation) was spread onto SMA (Himedia, India), Actinomycete Isolation Agar (AIA) (Himedia, India), and Humic Acid Vitamin B agar (HVA). Each medium was supplemented with nalidixic acid (50 *μ*g/mL) and cycloheximide (50 *μ*g/mL) to suppress the growth of Gram‐negative bacteria and fungi. The inoculated plates were incubated at 30°C for 2–4 weeks. Emerging colonies were carefully observed and selected based on typical actinomycete morphological characteristics.

### 2.3. Identification of Isolates

#### 2.3.1. Phenotypic Characterization

Actinomycete‐like colonies were picked, subcultured, and purified on International *Streptomyces* Project‐2 (ISP2) medium, and the plates were incubated at 30°C for 14 days. All isolates were analyzed for their morphological and biochemical characteristics for preliminary identification at the genus level. Morphological characteristics were assessed based on the color of aerial and substrate mycelia, production of diffusible pigment, and spore mass color, following the criteria described by Goodfellow and Haynes [17]. Acid production from various carbon sources was carried out following the method of Gordon et al. [18]. The basal inorganic nitrogen medium (g/L: (NH_4_)_2_HPO_4_, 1; KCl, 0.2; MgSO_4_.7H_2_O, 0.2; agar, 15) was adjusted to pH 7.0 prior to the addition of bromocresol purple. After autoclaving, 1% of each sterilized sugar was added to the medium. The isolated actinomycetes were cultured on slants containing these sugars for 7–14 days. A color change from purple to yellow indicated positive acid production. Biochemical tests, including citrate utilization, urease activity, indole production, methyl red (MR) and Voges‐Proskauer (VP) tests, and oxidase and catalase tests, were also performed on all isolates.

#### 2.3.2. Enzyme Activity Assays

Actinomycetes were first grown on ISP2 agar at 30°C for 7 days. A small portion of each colony was then inoculated onto enzyme‐specific media: chitin agar (g/L: 3 g colloidal chitin, 1.1 g Na_2_HPO_4_.2H_2_O, 0.7 g KH*_2_* PO_4_, 0.2 g MgSO_4_.7H_2_O, 0.01 g FeSO_4_.7H_2_O, 0.01 g MnSO_4_.H_2_O, 18 g agar, pH 7.0) for chitinase; starch agar (g/L: 2 g soluble starch, 5 g beef extract, 3 g peptone, 20 g agar) for amylase; gelatin agar (g/L: 7 g gelatin, 3 g beef extract, 5 g peptone, 20 g agar) for gelatinase; Tween agar (g/L: 10 g peptone, 5 g NaCl, 0.1 g CaCl_2_.2H_2_O, 10 mL Tween 80, 20 g agar) for lipase; and cellulose agar (g/L: 0.5 g (NH_4_)_2_SO_4_, 1 g K_2_HPO_4_, 0.2 g MgSO_4_, 0.5 g KCl, 0.1 g CaCl_2_, 0.5 g yeast extract, 10 g carboxymethyl cellulose (CMC), 18 g agar) for cellulase activity. Plates were incubated at 30°C for 7 days, and enzyme production was determined by the formation of hydrolysis zones around the colonies. Chitinase was indicated by clear halos on chitin agar. Amylase activity was detected by flooding starch agar with iodine solution, where clear zones indicated starch hydrolysis. Gelatinase activity was detected on gelatin agar plates by flooding the plates with mercuric chloride solution after incubation. Clear zones around colonies indicated gelatin hydrolysis. Lipase activity on Tween agar was indicated by the formation of an opaque precipitation zone around colonies. Cellulase activity was detected by staining plates with 0.1% Congo red followed by destaining with 1 M NaCl, where clear zones indicated cellulose degradation.

#### 2.3.3. DNA Amplification, Sequencing, and Phylogenetic Analysis

Total genomic DNA was extracted from cells cultivated in Glucose Yeast Extract (GYE) broth using a genomic isolation kit (Vivantis Technologies Sdn Bhd, Malaysia). The 16S rRNA genes were amplified and sequenced using primers 27F (5 ^′^‐AGA GTT TGA TCC TGG CTC AG‐3 ^′^) and 1525r (5 ^′^‐AAA GGA GGT GAT CCA GCC‐3 ^′^), as previously reported by Tatar [19] (U2Bio, Thailand). The obtained sequences were analyzed and edited using SeqScanner 2 and the BioEdit program [20]. Pairwise sequence similarities (%) of the 16S rRNA gene between the isolates and type strains were determined using the EzBioCloud server (http://eztaxon-e.ezbiocloud.net/) [21, 22]. Phylogenetic analysis based on 16S rRNA gene sequences was performed using the neighbor‐joining (NJ) method [23] in the Molecular Evolutionary Genetics Analysis (MEGA) software Version 11.0.13 [24].

### 2.4. Screening for Antagonistic Effect Against Pathogenic Organisms of Isolates

The antimicrobial activity of isolated actinomycete strains was qualitatively assessed using the perpendicular streak method [25, 26]. Each actinomycete isolate was streaked along the diameter of an agar plate and incubated at 30°C for 7 days. The selected test pathogens included *Bacillus cereus* ATCC 11778, *Bacillus subtilis* ATCC 6633, *Escherichia coli* ATCC 25922, *Salmonella* Typhimurium ATCC 14028, *Klebsiella pneumoniae* ATCC 13883, *Shigella sonnei* ATCC 25931, *Staphylococcus aureus* ATCC 29213, *Staphylococcus epidermidis* ATCC 12228, *Pseudomonas aeruginosa* ATCC 27853, *Enterococcus faecalis* ATCC 29212, *Enterococcus faecium* ATCC 35667, *Klebsiella aerogenes* ATCC 13048 (formerly *Enterobacter aerogenes*), *Listeria monocytogenes* ATCC 7644, and *Candida albicans* ATCC 10231. Pure cultures of the test bacteria and yeast were inoculated in Tryptic Soy Broth (TSB) and Yeast Malt (YM) broth, respectively, and incubated at 37°C for 24 h. The cultures were then adjusted to a turbidity equivalent to 0.5 McFarland (~1.5 × 10^8^ cells/mL), and streaked perpendicular to the actinomycete isolates on the agar plates. The plates were further incubated at 37°C for 24 h. Antimicrobial activity was determined by observing and measuring the inhibition zones formed between the actinomycete growth and the test pathogens.

### 2.5. Production of Crude Extract

Based on the results of the primary screening, isolate NRAIS18 was selected for secondary metabolite production. The isolate was inoculated into ISP2 broth and incubated at 30°C for 7 days under shaking conditions (150 rpm). After incubation, the culture broth was subjected to solvent extraction. Ethyl acetate was selected because it is a semipolar solvent widely used for recovering extracellular secondary metabolites from actinomycete fermentation broths and has been reported to produce extracts with the highest antimicrobial activity, suggesting that active metabolites preferentially partition into this solvent [27–29]. An equal volume of ethyl acetate (1:1, v/v) was added to the culture broth and vigorously shaken for 30 min. The extraction was repeated three times to maximize metabolite recovery. The combined organic layers were pooled and evaporated under reduced pressure (180–200 mbar) using an R‐100 rotary evaporator (Buchi, Flawil, Switzerland) at 40°C until dryness. The crude extract was then redissolved in dimethyl sulfoxide (DMSO) to the desired concentration for subsequent antimicrobial and chemical profiling assays.

### 2.6. Testing the Antimicrobial Activity of the Potent Strain Using the Agar Well Diffusion Method

The antimicrobial activity of the crude extract was assessed using the agar well diffusion method, following the recommended instruction of the Clinical and Laboratory Standards Institute (CLSI). Wells with a diameter of 6 mm were aseptically punched into the agar plate using sterile cork borers. Each test organism was adjusted to a turbidity equivalent to a 0.5 McFarland standard and evenly swabbed onto the agar surface. Each well was filled with 100 *μ*L of the crude extract (final concentration: 20 mg/mL). A well containing 20% DMSO was used as the negative control, corresponding to the final solvent concentration used to dissolve the crude extract and ensuring identical solvent conditions between the control and tested samples. The diameter of the inhibition zones was measured after 24 h of incubation at 37°C. All experiments were performed in triplicate.

### 2.7. LC‐MS Analysis

LC‐MS analysis was performed using an Agilent HPLC system equipped with a Poroshell 120 EC‐C18 column (100 × 2.1 mm, 2.7 *μ*m, Agilent, United States) maintained at 50°C. A 10‐*μ*L aliquot of crude extract, diluted to 20 mg/mL in 50% methanol, was injected. The mobile phase consisted of Solvent A (deionized water) and Solvent B (acetonitrile), both containing 0.1% (v/v) formic acid. Gradient elution was applied as follows: 55%–75% B from 10.5–12.5 min, followed by 100% B from 14.0–17.0 min, at a flow rate of 0.4 mL/min. Mass spectrometry was carried out on an Agilent LC‐QTOF 6545XT with an electrospray ionization (ESI) source using Jet Stream technology. Analysis was performed in both positive (20 eV) and negative (10 eV) ionization modes. Instrument settings included a drying gas flow of 13 L/min at 325°C, nebulizer pressure of 45 psi, sheath gas temperature of 275°C (12 L/min), capillary voltages of 4000 (positive) and 3000 V (negative). Mass ranges were set to m/z 40–1700 (MS1) and m/z 25–1000 (MS2) (Figure S1). Data processing was performed using MS‐DIAL Version 5.3. Metabolite identification was performed by spectral matching of ESI (+/−) MS/MS spectra against the MS‐DIAL public databases, including the Fiehn/Vaniya Natural Product Database, and the BMDMS‐NP library [30]. An identification scores greater than 0.7 was used as the threshold for acceptable spectral matching. Since authentic reference standards were not used for confirmation in this study, all compound annotations were considered putative and assigned based on spectral similarity and fragmentation pattern analysis.

### 2.8. Identification of Volatile Bioactive Compounds by GC‐MS

The chemical compounds present in the crude extract were identified by GC‐MS analysis, following the method described by Tedsree et al. [31]. The analysis was performed using a GC Agilent 6890 system coupled with an MS Hewlett 5973 detector. A 20‐min isothermal run was employed, with the injection port temperature maintained at 250°C and a temperature ramp rate of 5°C per minute. Helium was used as the carrier gas at a flow rate of 1 mL/min. The stationary phase was an HP‐5MS capillary column (30 m × 0.32 mm × 0.25 *μ*m), and the injection volume was 1 *μ*L. The detected compounds were tentatively identified by comparing their mass fragmentation patterns with those in the NIST mass spectral library. Only compounds with similarity scores ≥ 70% were considered for annotation. As authentic reference standards were not used in this study, the identified compounds were regarded as putative based on spectral similarity.

### 2.9. Genome De Novo Sequencing, Assembly, and Annotation

The genome of strain NRAIS18 was assembled from high‐quality sequencing reads using Unicycler v0.5.0. To determine its phylogenetic position, a tree was constructed based on genome composition comparisons with closely related type strains using the TYGS web server [32]. Comprehensive genome annotation and metabolic pathway reconstruction were performed using the RASTk annotation pipeline integrated into the PATRIC platform [33]. Moreover, antiSMASH was used to identify putative secondary metabolite biosynthetic gene clusters (BGCs) [34]. GenBank accession number for whole genome sequence of strain NRAIS18 is JBNBPQ000000000.

### 2.10. Genome‐Based Taxonomy and Phylogenomics

Species‐level identification of strain NRAIS18 was performed using digital DNA–DNA hybridization (dDDH) analysis via the Genome‐to‐Genome Distance Calibrator (GGDC) Version 2.1 [35]. Genomic relatedness was further assessed using average nucleotide identity (ANI) [22, 36]. Whole genome‐based taxonomic analysis was conducted by submitting the sequence data to the TYGS platform (https://tygs.dsmz.de; [32]).

## 3. Results

### 3.1. Isolation and Identification of Isolates

**Figure 1:**
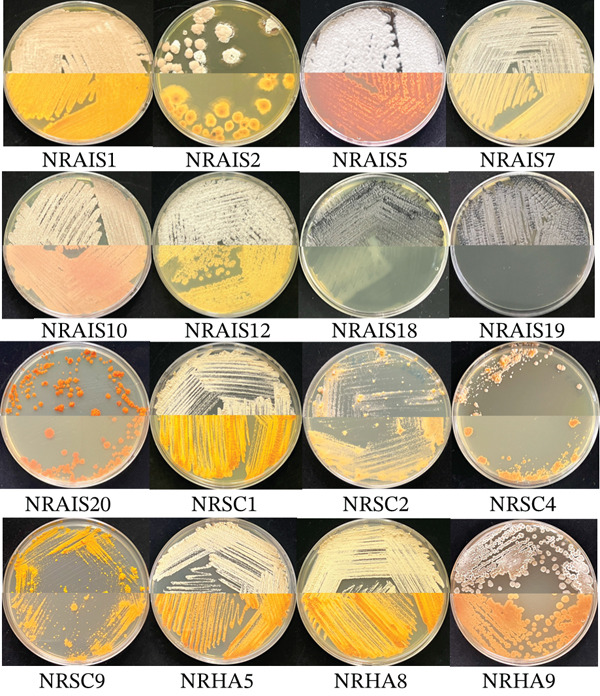
Morphological appearance of actinobacteria cultured on ISP2 at 30°C for 2 weeks. The upper half on each plate exhibits the front side of the culture, and the lower half shows the back side of each culture.

**Figure 2:**
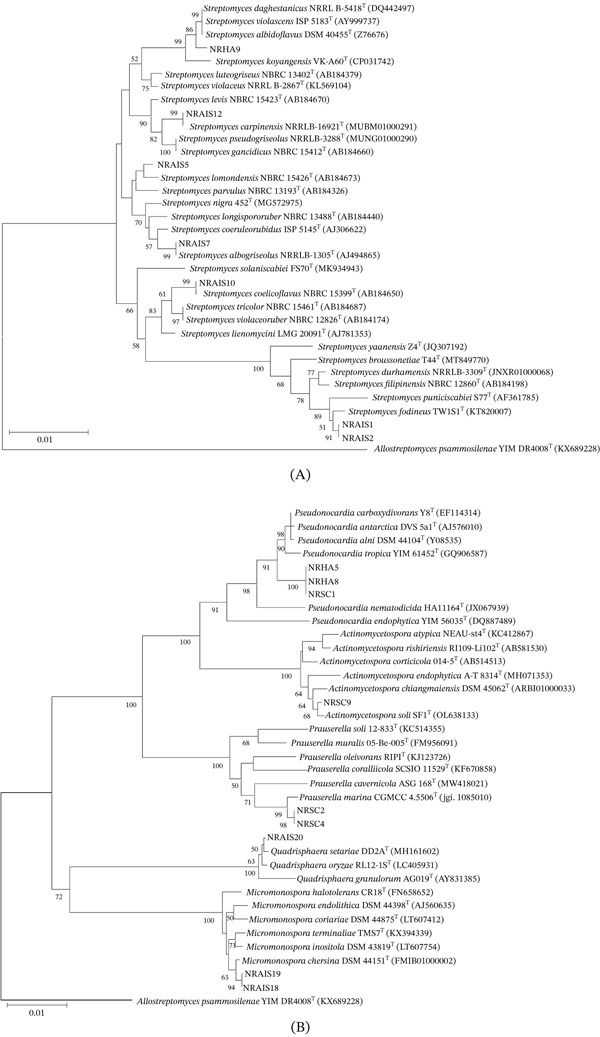
Neighbor‐joining (NJ) phylogenetic trees based on 16S rRNA gene sequences of actinomycetes isolated from *Mitragyna speciosa* leaves: (A) The *Streptomyces* group and (B) the non‐*Streptomyces* group, alongside related taxa. *Allostreptomyces psammosilenae* YIMDR4008^T^ was used as the outgroup. Bootstrap values ≥ 50% (based on 1000 bootstrap replicates) are indicated at branch nodes. The scale bar represents 0.01 substitutions per nucleotide position.

A total of 16 actinomycetes isolates were obtained from surface‐sterilized and nonsterilized *M. speciosa* leaves. Of these, nine isolates were recovered from AIA, four from SMA, and three from HVA. After 2–4 weeks of incubation, colonies appeared in white, orange, yellow, black, and brownish‐white colors (Figure 1). Based on phylogenetic trees constructed from 16S rRNA gene sequences (Figure 2) and phenotypic characteristics, the isolates were classified into six genera (Table 1): *Streptomyces* (Group I, seven isolates), *Micromonospora* (Group II, two isolates), *Quadrisphaera* (Group III, one isolate), *Pseudonocardia* (Group IV, three isolates), *Prauserella* (Group V, two isolates), and *Actinomycetospora* (Group VI, one isolate). All isolates showed 99.17%–100% 16S rRNA gene sequence similarity to their closely related species.

**Table 1:** Molecular identification of actinomycetes isolated from the leaves of medicinal *Mitragyna speciosa* Korth. based on 16S rRNA gene sequence analysis.

**Source**	**Strain no.**	**Closely related species**	**% Similarity**	**Accession no.**
Endophyte	NRAIS1	*Streptomyces durhamensis* NRRL B‐3309^T^	99.45	PV483699
Endophyte	NRAIS2	*S. durhamensis* NRRL B‐3309^T^	99.45	PV483712
Endophyte	NRAIS5	*Streptomyces lomondensis* NBRC 15426^T^	99.65	PV483710
Endophyte	NRAIS7	*Streptomyces albogriseolus* NRRL B‐1305^T^	100.00	PV483709
Endophyte	NRAIS10	*Streptomyces coelicoflavus* NBRC 15399^T^	99.17	PV483700
Endophyte	NRAIS12	*Streptomyces carpinensis* NRRL B‐16921^T^	100.00	PV483708
Endophyte	NRAIS18	*Micromonospora chersina* DSM 44151^T^	99.77	PV483698
Endophyte	NRAIS19	*M. chersina* DSM 44151^T^	99.77	PV483702
Endophyte	NRAIS20	*Quadrisphaera setariae* DD2A^T^	99.85	PV483701
Epiphyte	NRSC1	*Pseudonocardia carboxydivorans* Y8^T^	99.17	PV483705
Epiphyte	NRSC2	*Prauserella marina* CGMCC 4.5506^T^	99.65	PV483697
Epiphyte	NRSC4	*P. marina* CGMCC 4.5506^T^	99.65	PV483704
Epiphyte	NRSC9	*Actinomycetospora soli* SF1^T^	99.72	PV483703
Epiphyte	NRHA5	*P. carboxydivorans* Y8^T^	99.17	PV483707
Epiphyte	NRHA8	*P. carboxydivorans* Y8^T^	99.17	PV483713
Epiphyte	NRHA9	*Streptomyces albidoflavus* DSM 40455^T^	99.79	PV483706

Group I consisted of seven *Streptomyces* isolates, all of which formed white aerial mycelia with spore chains, except for isolate NRAIS10, which exhibited bluish gray aerial mycelium. The substrate mycelia were yellowish‐brown, brown, or reddish‐orange (NRAIS10). Based on 16S rRNA gene sequences and phylogenetic analysis, isolates NRAIS1 and NRAIS2 were most closely related to *S. durhamensis* NRRL B‐3309^T^ with 99.45% sequence similarity. The isolates NRAIS5, NRAIS7, NRAIS10, and NRAIS12 were closely related to *S. lomondensis* NBRC 15426^T^ (99.65%), *S. albogriseolus* NRRL B‐1305^T^ (100%), *S. coelicoflavus* NBRC 15399^T^ (99.17%), and *S. carpinensis* NRRL B‐16921^T^ (100%), respectively. Additionally, isolate NRHA9 (from nonsterilized leaves) was closely related to *S. albidoflavus* DSM 40455^T^ (99.79%).

Group II contained two isolates that produced single spore on the substrate mycelia. Colonies grown on ISP2 agar were initially pale orange and turned olive‐black after sporulation. Isolates NRAIS18 and NRAIS19 were closely related to *Micromonospora chersina* DSM 44151^T^, with 99.77% sequence similarity.

Group III included one isolate whose colonies grown on ISP2 were orange and nonspore forming. Isolate NRAIS20 was most closely related to *Quadrisphaera setariae* DD2A^T^, with 99.85% sequence similarity.

Group IV contained three isolates that produced white aerial and yellowish‐brown substrate mycelia. The aerial mycelia fragmented into rod‐shaped spores. Isolates NRSC1, NRHA5, and NRHA8 were closely related to *Pseudonocardia carboxydivorans* Y8^T^, with 99.17% sequence similarity.

Group V had two isolates with white aerial mycelia and moderately reddish‐brown substrate mycelium on ISP2 agar. Isolate NRSC2 and NRSC4 were closely related to *Prauserella marina* CGMCC 4.5506^T^, with 99.65% sequence similarities.

Group VI included one isolate. Colonies of NRSC9 were yellow and white, with powdery spores on the surface of the colonies. Isolate NRSC9 was closely related to *Actinomycetospora soli* SF1^T^, with 99.72% sequence similarity.

The biochemical characteristics, including enzyme activities, are summarized in Table 2. All isolates tested positive for catalase and negative for oxidase, indole, and the methyl red (MR) test. Urease activity was positive in some strains: NRAIS2 and NRHA9, with weak activity observed in NRSC9. Only strain NRSC9 tested positive for citrate utilization. All isolates produced acid from glucose, galactose, fructose, and xylose. However, strains NRSC1, NRHA5, and NRHA8 (identified as *P. carboxydivorans*); NRSC2 and NRSC4 (identified as *P. marina*); and NRSC9 (identified as *A. soli*) did not produce acid from lactose and soluble starch. Amylase activity was observed only in strains capable of acid production from soluble starch. Only strain NRAIS20 lacked lipase activity. All strains exhibited gelatinase activity. Some *Streptomyces* strains, along with NRAIS18 and NRAIS19 (identified as *M. chersina*), showed cellulase and chitinase activities.

**Table 2:** Phenotypic, biochemical, and enzymatic characterization of actinobacteria isolated from the leaves of the medicinal plant *Mitragyna speciosa* Korth.

Characteristic	NRAIS1	NRAIS2	NRAIS5	NRAIS7	NRAIS10	NRAIS12	NRAIS18	NRAIS19	NRAIS20	NRSC1	NRSC2	NRSC4	NRSC9	NRHA5	NRHA8	NRHA9
Urease activity	−	+	+	−	−	−	w	−	−	−	−	−	−	−	−	+
Citrate utilization	−	−	−	−	−	−	−	−	−	−	−	−	+	−	−	−
Indole	−	−	−	−	−	−	−	−	−	−	−	−	−	−	−	−
MR	−	−	−	−	−	−	−	−	−	−	−	−	−	−	−	−
VP	−	−	−	−	+	−	+	+	−	−	−	−	−	−	−	−
Catalase	+	+	+	+	+	+	+	+	+	+	+	+	+	+	+	+
Oxidase	−	−	−	−	−	−	−	−	−	−	−	−	−	−	−	−
Acid production																
Glucose	+	+	+	+	+	+	+	+	+	+	+	+	+	+	+	+
Lactose	+	+	+	+	+	+	+	+	−	−	−	−	−	−	−	−
Galactose	+	+	+	+	+	+	+	+	w	+	+	+	−	+	+	+
Sucrose	+	+	+	−	−	−	+	+	−	+	−	+	w	+	+	−
Maltose	+	+	+	+	+	+	+	+	−	w	+	−	w	−	−	+
Fructose	+	+	+	+	+	+	+	+	w	+	w	+	+	+	+	+
Mannitol	+	+	+	+	+	+	−	−	−	w	−	w	−	+	+	w
Xylose	+	+	+	+	+	+	+	+	+	+	+	+	w	+	+	+
Starch	+	+	+	+	+	+	+	+	−	−	−	−	−	−	−	+
Enzyme activity																
Amylase	+	+	+	+	+	+	+	+	−	−	−	−	−	−	−	+
Lipase	+	+	+	+	+	+	+	+	−	+	+	+	+	+	+	+
Caseinase	+	+	−	−	+	+	+	+	−	−	+	−	+	−	−	+
Gelatinase	+	+	+	+	+	+	+	+	+	+	+	+	+	+		+
Cellulase	−	−	+	+	+	−	+	+	−	−	−	−	−	−	−	−
Chitinase	−	+	+	−	+	−	+	+	−	−	−	−	−	−	−	−

*Note:* “+” denotes positive reaction; “w” refers to weakly positive reaction; and “−” indicates negative reaction. Positive and weakly positive reactions indicate the presence of enzymatic or biochemical activity, whereas negative reactions indicate no detectable activity under the test conditions.

### 3.2. Screening of Antimicrobial Activity of Obtained Isolates

A total of 16 pure isolates were subjected to preliminary antimicrobial assay using the perpendicular streak method. Seven isolates (five *Streptomyces* strains and two *Micromonospora* strains) showed activity against at least one of the 13 pathogenic bacteria, but none exhibited antagonistic activity against *C. albicans* ATCC 10231 (Table S1, Figure S2). Owing to its superior antimicrobial efficacy, as evidenced by the largest inhibition zone diameter, and its broader spectrum of activity—demonstrating effectiveness against a number of tested microorganisms—NRAIS18 was selected for further bioactivity investigations.

### 3.3. Antimicrobial Activity of *M. chersina* NRAIS18 Crude Extract

The crude extract from strain NRAIS18 was evaluated for antimicrobial activity using the agar well diffusion method. At a final concentration of 20 mg/mL, the extract showed the largest inhibition zone against *L. monocytogenes* ATCC 7644, followed by *P. aeruginosa* ATCC 27853 (Table 3, Figure S2). In contrast, smaller inhibition zones were observed against Gram‐negative bacteria, including *E. coli* ATCC 25922, *S. sonnei* ATCC 25931, and *Salmonella* Typhimurium ATCC 14028.

**Table 3:** Antimicrobial activity of the crude extract from *M. chersina* NRAIS18 against various Gram‐positive and Gram‐negative bacteria, indicating its inhibitory effects on different bacterial pathogens.

Tested microorganisms	Inhibition zone (mm)
*Bacillus cereus* ATCC 11778	20.50 ± 0.87C
*Bacillus subtilis* ATCC 6633	19.33 ± 0.58C
*Staphylococcus aureus* ATCC 29213	19.00 ± 0.87C
*Staphylococcus epidermidis* ATCC 12228	19.33 ± 0.29C
*Escherichia coli* ATCC 25922	11.50 ± 0.50E
*Salmonella* Typhimurium ATCC 14028	12.17 ± 0.29E
*Shigella sonnei* ATCC 25931	18.67 ± 2.08C
*Klebsiella pneumoniae* ATCC 13883	12.17 ± 0.29E
*Listeria monocytogenes* ATCC 7644	25.67 ± 0.76A
*Enterococcus faecium* ATCC 35667	19.83 ± 0.29C
*Enterococcus faecalis* ATCC 29212	20.17 ± 0.29C
*Klebsiella aerogenes* ATCC 13048	15.67 ± 0.29D
*Pseudomonas aeruginosa* ATCC 27853	23.33 ± 3.79B

*Note:* Data in the table are expressed as mean ± SD. Means with the same letters within each column are not significantly different at *p* < 0.05 level according to Duncan′s new multiple range test.

Abbreviation: SD, standard deviation.

### 3.4. Genomic Sequencing Analysis of *M. chersina* NRAIS18

**Figure 3:**
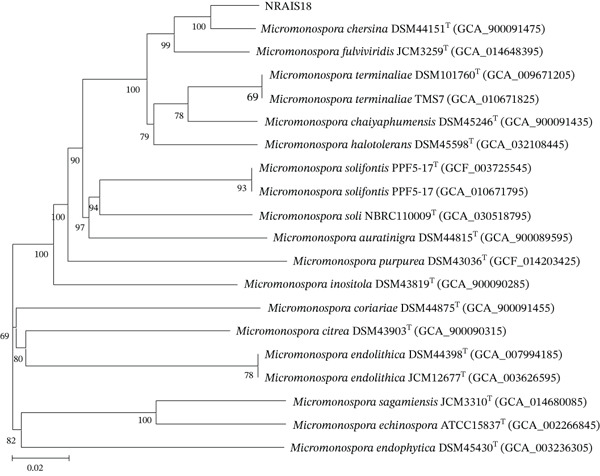
Phylogenomic tree of *Micromonospora chersina* NRAIS18 and related genera, based on whole genome sequences. Branch lengths are scaled using the Genome BLAST Distance Phylogeny (GBDP) distance formula d5. Numbers above the branches represent GBDP pseudo‐bootstrap support values derived from 100 replications.

Strain NRAIS18 exhibited a genome size of 6,826,327 bp (98 contigs, N50 value of 163,024) and a genomic G + C content of 73.7 mol%. Taxonomic analysis revealed the highest similarity to *M. chersina* DSM 44151^T^, based on an ANIb value of 96.17%, which exceeds the 95%–96% ANI threshold commonly used to delineate bacterial species [37] (Table S2). Additionally, this strain exhibited a dDDH value of 84.2% (formula d6) relative to *M. chersina* DSM 44151^T^, surpassing the 70% dDDH threshold for species delineation [38]. Phylogenomic analysis based on whole genome sequencing showed that strain NRAIS18 clustered closely with *M. chersina* DSM 44151^T^, sharing the same node in the phylogenetic tree (Figure 3).

### 3.5. Gene Function Annotation and Secondary Metabolism Gene Clusters

The antiSMASH analysis predicted 32 putative secondary metabolites BGCs in the strain NRAIS18. These clusters include terpene, polyketide synthase (PKS), nonribosomal peptide synthase (NRPS), NRPS‐like, lanthipeptide‐Classes I and III, RiPP‐like, and aminoglycoside (Table 4). The predicted secondary metabolites are associated with various antimicrobial and other bioactive compounds, such as loseolamycin, isorenieratene, enduracidin, desferrioxamine, SapB, lymphostin, and actinospectacin.

**Table 4:** Predicted biosynthetic gene clusters (BGCs) and their sequence similarity to characterized clusters in the MIBiG database.

Region	Position	Type	Most similar known cluster	Similarity score	Organism
1.1	266,437–292,931	Lanthipeptide‐Class‐I	Mechercharmycin	0.75	*Thermoactinomyces* sp.
3.1	101,710–123,858	Terpene	Isorenieratene	0.55	*Streptomyces griseus* subsp. *griseus* NBRC 13350
3.2	205,953–226,912	Terpene	Bagremycin A and Bagremycin B	0.45	*Streptomyces* sp. WK‐5344
4.1	256,669–296,280	T1PKS	Esperamicin	0.71	*Actinomadura verrucosospora*
5.1	167,102–208,160	T3PKS	Loseolamycin A1 and Loseolamycin A2	0.85	*Micromonospora endolithica*
8.1	56,122–163,607	NRPS‐like, NRPS, T3PKS	Enduracidin A and Enduracidin B	1.20	*Xenorhabdus nematophila* ATCC 19061
10.1	69,071–110,093	PKS‐like	Kolossin	0.63	*Photorhabdus laumondii* subsp. *laumondii* TTO1
12.1	132,933–180,458	NRPS, T1PKS	AM‐toxin	0.84	*Alternaria alternata*
15.1	79,906–109,678	NI‐siderophore	Desferrioxamine E	0.83	*Streptomyces* sp. ID38640
16.1	50,599–71,549	Terpene	Albaflavenone	0.52	*Streptomyces coelicolor* A3(2)
16.2	131,511–152,575	Terpene‐precursor	Isorenieratene	0.46	*Streptomyces argillaceus*
18.1	7445–79,957	T2PKS	Spore pigment	0.62	*Streptomyces avermitilis*
20.1	122,493–144,371	Lanthipeptide‐class‐iii	SapB	0.77	*S. coelicolor* A3(2)
32.1	3029–28,971	Terpene, RiPP‐like	Lymphostin, Neolymphostinol B, Lymphostinol, and Neolymphostin B	0.31	*Salinispora arenicola* CNS‐205
34.1	19,355–62,164	NRPS	Icosalide A/B	0.76	*Burkholderia gladioli*
35.1	1–61,509	NRPS,T1PKS, NRPS‐like	Dimethylcoprogen	1.25	*A. alternata*
37.1	31,870–52,937	Terpene‐precursor	CDA1b, CDA2a, CDA2b, CDA3a, CDA3b, CDA4a, and CDA4b	0.39	*Streptomyces melanosporofaciens*
40.1	18,614–33,351	NAGGN	Scytodecamide	0.45	*Scytonema* sp. UIC 10036
43.1	7402–28,202	Amglyccycl	Actinospectacin	0.49	*Streptomyces griseofuscus*
47.1	1456–41,975	NRPS‐like, T1PKS, 2dos	Quinolidomicin A	1.32	*Aspergillus ochraceus*
55.1	746–22,077	Terpene	Bicornutin A1 and Bicornutin A2	0.55	*Xenorhabdus budapestensis*
58.1	1–18,640	T1PKS	Sceliphrolactam	0.76	*A. ochraceus*
59.1	1–16,099	T1PKS	Halstoctacosanolide A	0.65	*Streptomyces albus*
60.1	1–15,524	T1PKS	Griseochelin	0.72	*Pseudomonas syringae*
63.1	1–14,850	T1PKS	67‐121C	0.65	*P. syringae*
71.1	1–10,459	T1PKS	Apoptolidin	0.64	*P. syringae*
72.1	1–10,234	T1PKS	Heronamide A/B/C/D/E/F	0.65	*Fusarium graminearum* PH‐1
73.1	1–9959	T1PKS	Nigericin	0.73	*Parastagonospora nodorum*
74.1	1–9252	T1PKS	Quinolidomicin A	0.64	*A. ochraceus*
76.1	1–6025	T1PKS	Alternariol	0.62	*P. nodorum* SN15
77.1	1–4300	T1PKS	Asperlactone	0.57	*A. ochraceus*
78.1	1–4167	T1PKS	Asperlactone	0.64	*A. ochraceus*

### 3.6. Metabolic Profiling of the Crude Extract From Strain NRAIS18 by LC‐MS Analysis

The chemical analysis of the ethyl acetate crude extract from strain NRAIS18 revealed the presence of various groups of bioactive compounds, including benzimidazoles, isoflavones, pyranones and derivatives, chromones, alkaloids, and amino acids (Table 5). These compounds have been reported to possess antimicrobial activity. The major metabolites identified were 5,6‐Dimethylbenzimidazole, daidzein (a soy‐derived product), maltol, harman, norharman, and saponins (soyasaponin Bb). Moreover, some compounds in the macrolactam group, such as nocardamine (desferrioxamine E), were classified as exhibiting indirect antimicrobial activity, primarily through their role as siderophores.

**Table 5:** Putative secondary metabolites with reported antimicrobial activity detected in the crude extract of *M. chersina* NRAIS18 based on LC‐MS analysis and tentative identification through comparison of mass spectral data with references databases.

Group	Metabolite	Ion type	Measured (m/z)	Molecular formula	Total score	Retention time	Arbitrary unit (a.u.)	Antimicrobial activity	Reference
Benzimidazoles	5,6‐Dimethyl benzimidazole	[M + H]	147.09	C_9_H_10_N_2_	1.80	3.799	18,344,807	5,6‐Dimethylbenzimidazole produced by *Streptomyces* sp. KN37, exhibited good inhibitory effects on *R. solani* and *E. amylovora.*	[39]
Isoflavones	Daidzein	[M−H]^−^	253.06	C_15_H_10_O_4_	1.73	6.332	13,184,503	Daidzein potently inhibited *S. aureus*, MRSA, and *Vibrio harveyi*, supporting its role as an antimicrobial scaffold.	[40]
Pyranones and derivatives	Maltol	[M + H]	127.04	C_6_H_6_O_3_	1.41	3.019	9,065,745	*Streptomyces* sp. E2 exhibited broad‐spectrum antibacterial activity, with maltol being the most prominent component of its crude extract.	[41]
Chromones	(2R)‐5‐methoxy‐2‐methyl‐2,3,8,9‐Tetrahydrofuro[2,3‐h] chromen‐4‐one	[M−H]^−^	233.08	C_13_H_14_O_4_	1.30	5.474	7,267,080	A 4H‐chromen‐4‐one derivative from *Streptomyces ovatisporus* S4702^T^ showed strong antibacterial activity against *B. subtilis* and *Micrococcus luteus*, with MICs as low as 0.25 *μ*g/mL.	[42]
Alpha amino acids and derivatives	Cyclo (Leu‐Pro) or diketopiperazine	[M + H]^+^	211.15	C_11_H_18_N_2_O_2_	1.52	4.557	8,684,109	Diketopiperazines such as cyclo(Leu‐Pro) have been reported to be produced by *Micromonospora* sp. and possess antibacterial activity.	[43]
	Betaine	[M + H]^+^	118.09	C_5_H_11_NO_2_	1.67	0.687	6,941,436	A study on betaine esters (C10‐C18) found the tetradecyl derivative effectively killed *Salmonella* Typhimurium (99.99% in 3 minutes at pH 6), indicating its potential as a disinfectant for food and surfaces.	[44]
Flavonols	Galangin	[M−H]^−^	269.05	C_15_H_10_O_5_	1.70	7.318	8,689,146	Galangin exhibited significant antibacterial activity against ancomycin‐intermediate *S. aureus* strains, highlighting its potential as a viable alternative therapeutic agent.	[45]
Beta carbolines	Norharman	[M + H]^+^	169.08	C_11_H_8_N_2_	1.74	4.234	7,681,256	Norharman and its derivatives, including the dimer and N2‐methyl forms, exhibit potent antibacterial activity against *S. aureus.*	[46]
Harmala alkaloids	Harman	[M + H]^+^	183.10	C_12_H_10_N_2_	1.65	4.609	6,714,383	Harman exhibited antimicrobial activity, achieving 17.9% growth inhibition against *E. coli* and 15.8% against *Aspergillus niger*.	[46]
Dialkyl phosphates	Bis (2‐ethylhexyl) phosphate	[M−H]^−^	321.22	C_16_H_35_O_4_P	1.68	13.099	6,685,914	*Lactiplantibacillus plantarum* BCH‐1 produced DEHP, demonstrating antibacterial activity against *E. coli* and *S. aureus.*	[47]
Macrolactams	Ferrioxamine E	[M + H]^+^	654.27	C_27_H_45_FeN_6_O_9_	1.21	3.970	4,586,132	Hydroxamate‐type siderophore	[48]
Pyrroloindoles	Roquefortine C	[M−H]^−^	388.19	C_22_H_23_N_5_O_2_	0.89	6.28	3,951,230	Roquefortine C preferentially inhibited hemin‐positive Gram‐positive bacteria, with Gram‐negative cells remaining resistant.	[49]
Macrolactams	Desferrioxamine E	[M + Cl]^−^	635.33	C_27_H_48_N_6_O_9_	0.99	5.133	2,887,891	Hydroxamate‐type siderophore	[48]
Triterpene saponins	Soyasaponin Bb	[M−H]^−^	941.51	C_48_H_78_O_18_	1.62	9.58	2,700,282	Soyasaponin I, produced by *Streptomyces,* has been reported to possess antimicrobial activity against *E. coli* and *C. albicans*.	[50, 51]
Diterpenoids	Isorenieratene	[M + H]^+^	528.37	C_40_H_48_	0.88	9.39	45,390	Isorenieratene is a carotenoid‐related metabolite associated with terpenoid biosynthetic pathways in members of the genus *Micromonospora*	[52]

### 3.7. Identification of Bioactive Compounds of Strain NRAIS18 Using GC‐MS Analyses

The chemical composition of the ethyl acetate crude extract obtained from strain NRAIS18 was analyzed using GC‐MS. A total of 22 compounds were detected in the extract, of which 15 compounds showed a similarity index higher than 70% based on library matching. Among these, a literature review confirmed that 13 of these compounds have been previously reported possessing antimicrobial activity (Table 6). The predominant compounds were 1,2,3,4‐tetrahydro‐6,7‐dihydroxy‐3‐isoquinolinecarboxylic acid, cyclotrisiloxane, hexamethyl, benzeneethanol, 2,5,5‐trimethyl‐1,3‐cyclohexanedione, and diethyldithiophosphinic acid.

**Table 6:** Putative volatile compounds with reported antimicrobial activity detected in the ethyl acetate crude extract of *M. chersina* NRAIS18 based on GC‐MS analysis.

RT	Compound name	MW	MF	Similarity	Reported bioactivity	References
9.471	1,2,3,4‐Tetrahydro‐6,7‐dihydroxy‐3‐isoquinolinecarboxylic acid	209	C_10_H_11_NO_4_	83	Antibacterial activity	[53]
10.746	Octamethylcyclotetrasiloxane	296	C_8_H_24_O_4_Si_4_	79	Antibacterial activity	[54]
12.450	2‐Furanol, tetrahydro‐2‐methyl	102	C_5_H_10_O_2_	80	No activity reported	—
13.352	Cyclotrisiloxane, hexamethyl	222	C_6_H_18_O_3_Si_3_	74	Antibacterial activity, antifungal activity	[55]
15.709	Benzeneethanol	122	C_8_H_10_O	86	Antibacterial activity	[56]
15.857	4H‐Pyran‐4‐one, 3‐hydroxy‐2‐methyl or Maltol	126	C_6_H_6_O_3_	79	Antibacterial activity, antifungal activity	[41]
18.631	Benzeneacetic acid, methyl ester	150	C_9_H_10_O_2_	92	Antibacterial activity	[57]
21.113	Cyclotetrasiloxane, octamethyl	296	C_8_H_24_O_4_Si_4_	83	Antibacterial activity	[54]
21.638	2‐methoxy‐3,8‐dioxocephalotax‐1‐ene	327	C_18_H_17_NO_5_	99	Antibacterial activity	[58]
22.592	Isoamyl phenylacetate	206	C_13_H_18_O_2_	84	No activity reported	—
23.594	1H‐indole	117	C_8_H_7_N	80	Antimicrobial activity	[59]
25.254	Glycine	75	C_2_H_5_NO_2_	77	Antibacterial activity	[60]
47.033	2,5,5‐Trimethyl‐1,3‐cyclohexanedione	154	C_9_H_14_O_2_	73	Antibacterial activity	[61]
47.711	Diethyldithiophosphinic acid	154	C_4_H_11_PS_2_	99	Antibacterial activity, antifungal activity	[62]
48.773	Hexadecanoic acid (palmitic acid)	256	C_16_H_32_O_2_	83	Antifungal activity	[63]

## 4. Discussion

Actinomycetes isolated from medicinal plants are a recognized source of numerous biologically active secondary metabolites, which may be associated with the therapeutic properties of their host plants [64]. Long‐term symbiotic relationships between endophytes and plants can lead to the integration of endophytes into the metabolic pathways of plants, thereby enhancing their natural bioactivity or enabling the production of specific physiologically active molecules that resemble those naturally produced by the host plant [8]. In this study, seven *Streptomyces* isolates and nine non‐*Streptomyces* isolates were obtained from the leaves of the medicinal plant *M. speciosa* collected in Thailand. Nine isolates, including *S. durhamensis*, *S. lomondensis*, *S. albogriseolus*, *S. coelicoflavus*, *S. carpinensis*, *M. chersina*, and *Q. setariae*, were obtained from surface‐sterilized leaves. The remaining isolates, including *P. marina*, *A. soli*, *P. carboxydivorans*, and *S. albidoflavus,* were isolated from nonsurface‐sterilized leaves and are likely epiphyte actinomycetes. Within the phyllosphere of various plants, *Actinomycetota* can exist as both epiphytes and endophytes [65].

Various actinomycetes, including *Streptomyces* and *Micromonospora*, have previously been isolated from medicinal plants such as *Ficus benzamine*, *Clinacanthus siamensis*, *Artemisia annua*, and *Portulaca oleracea*, all of which possess antimicrobial bioactive compounds [66]. In this study, strain NRAIS18 exhibited notable antibacterial activity compared with other strains during primary antibiotic production screening. Based on 16S rRNA gene sequence and genomic analyses, strain NRAIS18 was identified as *M. chersina.* Although soil is generally recognized as the primary source for the recovery and isolation of *Micromonospora* [43], several studies—including the present one—have also reported their occurrence in plant leaves [67]. The ethyl acetate crude extract of this strain exhibited potential antibacterial activity against both Gram‐positive and Gram‐negative bacteria. This antagonistic activity was found to agree with the antiSMASH pipeline production of several BGCs. Genome mining and antiSMASH analysis of strain NRAIS18 revealed that the dominant predicted secondary metabolites—NRPS, PKS, T1PKS, and terpene—were consistent with those previously reported in *Micromonospora* spp. This finding confirms that the genus *Micromonospora* has significant potential for producing secondary metabolites, including many novel compounds with antimicrobial activity [43]. In addition, BGCs related to loseolamycin, isorenieratene, desferrioxamine, and SapB were identified in the genome of NRAIS18, aligning with previous reports based on physicochemical analyses and bioinformatics genome mining of *Micromonospora* sp. [52, 68–70]. Metabolomic analysis of the crude extract using LC‐MS further supported the genomic predictions by revealing a diverse array of secondary metabolites, several of which were consistent with BGCs predicted in the genome. Among them, the hydroxamate siderophore ferrioxamine E and desferrioxamine E were tentatively identified. Desferrioxamines are hydroxamate siderophores commonly produced by actinomycetes that facilitate iron acquisition under iron‐limited conditions and may contribute to microbial competition by restricting iron availability to competing microorganisms. Their metal‐chelating properties have also attracted interest for the therapeutic applications and antimicrobial strategies, including the siderophore‐mediated “Trojan horse” approach to enhance antibacterial efficacy [70]. In addition, LC‐MS analysis also suggested the presence of isorenieratene, an aromatic carotenoid consistent with the terpene‐related BGCs predicted in the genome, suggesting that the carotenoid biosynthesis pathway may be functionally expressed in strain NRAIS18. Carotenoid biosynthesis pathways, including those potentially leading to aromatic carotenoids such as isorenieratene, have been reported in several *Micromonospora* genomes, highlighting the metabolic diversity and ecological adaptability of this genus [52].

Interestingly, some detected compounds, such as daidzein, soyasaponin Bb, and galangin, are commonly regarded as plant‐derived metabolites. However, similar compounds have also been reported from actinomycetes and other endophytic microorganisms. For instance, the endophytic strain *Streptomyces* sp. SS52 has been reported to produce daidzein [71] and soyasaponin‐type compounds have also been detected from plant endophytic *Streptomyces* sp. YIM 56130 isolated from *Drymaria diandra* [50]. These findings support that endophytes associated with medicinal plants may produce metabolites resembling those of their host plants due to long‐term ecological interactions [8]. In addition to these metabolites, several compounds detected in the crude extract—including 5,6‐dimethyl‐benzimidazole, maltol, (2R)‐5‐methoxy‐2‐methyl‐2,3,8,9‐tetrahydrofuro[2,3‐h] chromen‐4‐one (Chromones), harman, norharman, and soyasaponin Bb—are known to be produced by actinomycetes such as *Streptomyces* and *Actinomadura.* These compounds have been reported to exhibit antimicrobial activity against pathogens including *E. coli*, *S. aureus*, *B. subtilis*, and *Micrococcus luteus* [39, 41, 42, 72]. Other compounds identified in the crude extract, such as the amino acids betaine, Cyclo (Leu‐Pro), and glycine, as well as the fatty acid palmitic acid, have also been reported to exhibit antimicrobial activity [43, 44, 63]. GC‐MS analysis revealed a high number of compounds in the extract that have been previously reported possessing antibacterial activity, including an isoquinoline carboxylic acid derivative [53] and benzeneethanol [56]. Some secondary metabolites identified in the crude extract have also been previously detected in plants. For instance, octamethylcyclotetrasiloxane has been found in *Polyalthia cinnamomea* leaves, and cyclotrisiloxane, hexamethyl has been reported in green algae. All of these compounds exhibit antibacterial activity against various pathogens, including *B. subtilis*, *E. coli*, *S. aureus*, *Salmonella* sp., *K. pneumoniae*, *P. aeruginosa*, and *Acinetobacter baumanii* [54, 55, 57]. Medicinal plant‐associated actinomycetes are valuable sources of various secondary metabolites, some of which resemble those produced by plants—such as flavonoids, phenolics, alkaloids, and triterpene saponins—and can serve as effective antibacterial compounds. In addition, microbial biotransformation by actinomycetes may modify plant‐related metabolites, generating new bioactive derivatives through enzymatic reactions [73]. These findings highlight the potential of plant‐associated actinomycetes as sources of bioactive metabolites. Future studies will focus on the isolation and purification of selected metabolites, followed by evaluation of their antimicrobial activity and determination of their minimum inhibitory concentrations (MICs).

## 5. Conclusion

Sixteen actinomycetes, including both *Streptomyces* and non‐*Streptomyces* strains, were isolated from the leaves of *M. speciosa.* These included *S. durhamensis*, *S. lomondensis*, *S. albogriseolus*, *S. coelicoflavus*, *S. carpinensis*, *S. albidoflavus*, *M. chersina*, *Q. setariae*, *P. marina*, *A. soli*, and *P. carboxydivorans*. Among them, *M. chersina* strain NRAIS18 demonstrated notable antibacterial activity against both Gram‐positive and Gram‐negative pathogens. The ethyl acetate crude extract of NRAIS18 exhibited the strongest inhibition against *L. monocytogenes*, followed by *P. aeruginosa.* Chemical analysis of the extract revealed the presence of various secondary metabolites, including flavonoids, chromones, beta‐carbolines, macrolactams, alkaloids, triterpene saponins, amino acids, and fatty acids—many of which are reported to contribute to its potent antibacterial activity. Therefore, *M. chersina* NRAIS18 shows strong potential as a source of antibacterial compounds for pharmacological applications.

**Nomenclature**
AIA: Actinomycete Isolation Agar
ANI: average nucleotide identity
BGCs: biosynthetic gene clusters
CLSI: Clinical and Laboratory Standards Institute
CMC: carboxymethyl cellulose
dDDH: digital DNA‐DNA hybridization
DMSO: dimethyl sulfoxide
ESI‐MS: electrospray ionization mass spectra
GC‐MS: Gas Chromatography‐Mass Spectrometry
GYE: Glucose Yeast Extract
GGDC: Genome‐to‐Genome Distance Calibrator
HVA: Humic Acid Vitamin B Agar
HPLC: High‐Performance Liquid Chromatography
ISP: International *Streptomyces* Project
LC‐MS: Liquid Chromatography‐Mass Spectrometry
MEGA: Molecular Evolutionary Genetics Analysis
MIC: minimum inhibitory concentration
MR: methyl red
NJ: neighbor joining
NRPS: nonribosomal peptide synthetase
PKS: polyketide synthase
RiPP: ribosomally synthesized and posttranslationally modified peptides
SMA: Starch M‐Protein Agar
T1PKS: type I polyketide synthase
TSB: Tryptic Soy Broth
YM: Yeast Malt
TYGS: Type (Strain) Genome Server
VP: Voges‐Proskauer

## Funding

This study was supported by Science Research and Innovation Fund (FF66‐P1‐074).

## Ethics Statement

The authors declare that this study did not involve human participants, animals, or identifiable personal data.

## Conflicts of Interest

The authors declare no conflicts of interest.

## Supporting information


**Supporting Information** Additional supporting information can be found online in the Supporting Information section. Table S1: Antimicrobial activities of the actinomycetes isolated from *M. speciosa*. leaves against tested organisms. Table S2: Genome characteristics and pairwise sequence similarities (%) of the 16S rRNA gene sequences of the strain NRAIS18 and the closely related species. Figure S1: Representative full‐scan electrospray ionization mass spectra (ESI‐MS) acquired in (A) positive (+) and (B) negative (−) ionization modes (m/z 40–1700) from the crude extract of strain NRAIS18 compared with the solvent blank. Prominent m/z features detected exclusively in the crude extract and absent in the blank indicate metabolite‐derived ions produced by strain NRAIS18. Figure S2: Representative images showing the antimicrobial activity of *M. chersina* NRAIS18. (A) Perpendicular streak assay used for preliminary screening of actinomycete isolates from *M. speciosa* leaves. (B) Agar well diffusion assay showing inhibition zones produced by the crude extract against the tested pathogens, including *Babillus cereus* ATCC 11778, *Staphylococcus aureus* ATCC 29213, *Pseudomonas aeruginosa* ATCC 27853, and *Candida albicans* ATCC 10231.

## Data Availability

The data that support the findings of this study are available from the corresponding authors upon reasonable request.
