# Supplementary material for: Antimicrobial Activity of Actinobacteria Isolated From Kratom (Mitragyna speciosa) Leaves: Secondary Metabolite Profiling and Genome Analysis of Micromonospora chersina NRAIS18
Source: Int J Microbiol. 2026 May 5;2026:3823241. doi: 10.1155/ijm/3823241 (PMC13140871; doi:10.1155/ijm/3823241)
Supplement: Supplementary file 1 — Supporting Information Additional supporting information can be found online in the Supporting Information section. Table S1: Antimicrobial activities of the actinomycetes isolated from M. speciosa. leaves against tested organisms. Table S2: Genome characteristics and pairwise sequence similarities (%) of the 16S rRNA gene sequences of the strain NRAIS18 and the closely related species. Figure S1: Representative full‐scan electrospray ionization mass spectra (ESI‐MS) acquired in (A) positive (+) and (B) negative (−) ionization modes (m/z 40–1700) from the crude extract of strain NRAIS18 compared with the solvent blank. Prominent m/z features detected exclusively in the crude extract and absent in the blank indicate metabolite‐derived ions produced by strain NRAIS18. Figure S2: Representative images showing the antimicrobial activity of M. chersina NRAIS18. (A) Perpendicular streak assay used for preliminary screening of actinomycete isolates from M. speciosa leaves. (B) Agar well diffusion assay showing inhibition zones produced by the crude extract against the tested pathogens, including Babillus cereus ATCC 11778, Staphylococcus aureus ATCC 29213, Pseudomonas aeruginosa ATCC 27853, and Candida albicans ATCC 10231. [file IJM-2026-3823241-s001.docx]

**Supplementary Table 1.** Antimicrobial activities of the actinomycetes isolated from *M. speciosa*. leaves against tested organisms

| Tested microorganisms | Antimicrobial activity (Inhibition distance, mm) | | | | | | | | | | | | | | | |
| --- | --- | --- | --- | --- | --- | --- | --- | --- | --- | --- | --- | --- | --- | --- | --- | --- |
|  | NRAIS1 | NRAIS2 | NRAIS5 | NRAIS7 | NRAIS10 | NRAIS12 | NRAIS18 | NRAIS19 | NRAIS20 | NRSC1 | NRSC2 | NRSC4 | NRSC9 | NRHA5 | NRHA8 | NRHA9 |
| *B. cereus* | +++ | +++ | - | - | +++ | - | +++ | ++ | - | - | - | - | - | - | - | - |
| *B. subtilis* | +++ | +++ | - | - | ++ | - | +++ | +++ | - | - | - | - | - | - | - | - |
| *S. aureus* | +++ | +++ | - | - | - | - | +++ | ++ | - | - | - | - | - | - | - | - |
| *S. epidermidis* | +++ | +++ | - | + | ++ | +++ | +++ | +++ | - | - | - | - | - | - | - | - |
| *E. coli* | +++ | ++ | - | - | - | - | + | + | - | - | - | - | - | - | - | - |
| *Salmonella* | ++ | ++ | - | - | - | - | + | + | - | - | - | - | - | - | - | - |
| *Shigella* | ++ | ++ | - | - | - | - | + | + | - | - | - | - | - | - | - | - |
| *Klebsiella* | - | - | - | - | - | - | + | + | - | - | - | - | - | - | - | - |
| *L. monocytogenes* | +++ | +++ | - | - | - | - | +++ | +++ | - | - | - | - | - | - | - | - |
| *Ent. faecium* | +++ | +++ | - | + | - | - | +++ | ++ | - | - | - | - | - | - | - | - |
| *Ent. faecalis* | +++ | +++ | - | - | - | - | +++ | +++ | - | - | - | - | - | - | - | - |
| *K. aerogenes* | +++ | +++ | - | - | - | - | + | + | - | - | - | - | - | - | - | - |
| *P. aeruginosa* | - | - | - | - | - | - | + | + | - | - | - | - | - | - | - | - |
| *C. albicans* | - | - | - | - | - | - | - | - | - | - | - | - | - | - | - | - |

*Note:* Antimicrobial activity was indicated as inhibition distance: +++, 26–35 mm; ++, 16–25 mm; +, 5–15 mm; -, no inhibition.

**Supplementary Table 2.** Genome characteristics and pairwise sequence similarities (%) of the 16S rRNA gene sequences of the strain NRAIS18 and the closely related species.

The draft genome sequences of the strain NRAIS18 was determined in this study, while genome sequences of other species were retrieved from the GenBank database.

| Species | Strain | DNA G+C content (%) | 16S rRNA gene similarity (%) | ANIb value (%) | dDDH value (%) |
| --- | --- | --- | --- | --- | --- |
| *Micromonospora chersina* | DSM 44151^T^ | 73.6 | 99.77 | 96.17 | 84.2 |
| *Micromonospora endolithica* | DSM 44398^T^ | 72.5 | 99.55 | 81.36 | 29.1 |
| *Micromonospora terminaliae* | TMS7^T^ | 73.4 | 99.40 | 91.24 | 62.2 |
| *Micromonospora inositola* | DSM 43819^T^ | 72.2 | 99.32 | 85.86 | 38.3 |
| *Micromonospora halotolerans* | DSM 45598^T^ | 73.3 | 99.32 | 91.35 | 60.7 |
| *Micromonospora coriariae* | DSM 44875^T^ | 71.8 | 99.10 | 82.02 | 32.4 |

**Supplementary Figure 1.** Representative full-scan electrospray ionization mass spectra (ESI-MS) acquired in positive (+) (A) and negative (-) (B) ionization modes (m/z 40-1700) from the crude extract of strain NRAIS18 compared with the solvent blank. Prominent m/z features detected exclusively in the crude extract and absent in the blank indicate metabolite-derived ions produced by strain NRAIS18





**Supplementary Figure 2.** Representative images showing the antimicrobial activity of *M. chersina* NRAIS18. (A) Perpendicular streak assay used for preliminary screening of actinomycete isolates from *Mitragyna speciosa* leaves. (B) Agar well diffusion assay showing inhibition zones produced by the crude extract against the tested pathogens, including *Babillus cereus* ATCC 11778, *Staphylococcus aureus* ATCC 29213, *Pseudomonas aeruginosa* ATCC 27853, and *Candida albicans* ATCC 10231
